# The role of inflammatory and anti-inflammatory cytokines in the pathogenesis of human tegumentary leishmaniasis☆

**DOI:** 10.1016/j.cyto.2013.12.016

**Published:** 2014-01-30

**Authors:** Walker Nonato Oliveira, Luís Eduardo Ribeiro, Albert Schrieffer, Paulo Machado, Edgar M. Carvalho, Olívia Bacellar

**Affiliations:** a Serviço de Imunologia, Hospital Universitário Prof. Edgard Santos, Universidade Federal da Bahia, Salvador, Bahia, Brazil; b Instituto Nacional de Ciência e Tecnologia de Doenças Tropicais INCT-DT (CNPq/MCT), Salvador, Bahia, Brazil

**Keywords:** Human american tegumentary, leishmaniasis, *Leishmania braziliensis*, Immunopathogenesis, Regulatory cytokines

## Abstract

In tegumentary leishmaniasis caused by *Leishmania braziliensis*, there is evidence that increased production of IFN-γ, TNF-α and absence of IL-10 is associated with strong inflammatory reaction and with tissue destruction and development of the lesions observed in cutaneous leishmaniasis (CL) and mucosal leishmaniasis (ML). We evaluate the role of regulatory cytokines and cytokine antagonists in the down-regulation of immune response in *L. braziliensis* infection. Peripheral blood mononuclear cells from CL and ML were stimulated with soluble *Leishmania* antigen in the presence or absence of regulatory cytokines (IL-10, IL-27 and TGF-β) or antagonists of cytokines (α-TNF-α and α-IFN-γ). Cytokines production (IL-10, IL-17, TNF-α and IFN-γ) was measured by ELISA. IL-10 and TGF-β downmodulate TNF-α and IL-17 production, whereas IL-27 had no effect in the production of TNF-α, IFN-γ and IL-17 in these patients. Neutralization of TNF-α decreased IFN-γ level and the neutralization of IFN-γ decreased TNF-α level and increased IL-10 production. This study demonstrate that IL-10 and TGF-β are cytokines that appear to be more involved in modulation of immune response in CL and ML patients. IL-10 might have a protective role, since the neutralization of IFN-γ decreases the production of TNF-α in an IL-10-dependent manner.

## 1. Introduction

Type 1 immune responses and the IFN-γ and TNF-α produced by these responses are important to prevent growth of intracellular microbial agents and control dissemination of infections with intracellular agents like *Leishmania* [1,2]. However, in tegumentary leishmaniasis caused by *Leishmania braziliensis*, there is evidence that increased production of IFN-γ and TNF-α is associated with increased inflammatory reaction and development of cutaneous and mucosal ulcers, despite low numbers of detectable parasites in lesions using conventional techniques [3,4]. Moreover, lymphocyte activation, proliferation and cytokine secretion upon stimulation with *Leishmania* antigens are higher in mucosal leishmaniasis compared with cutaneous leishmaniasis [1,5], which corresponds with the severity of tissue destruction in mucosal leishmaniasis. IL-17 is also expressed at a higher level in the peripheral blood and tissues of mucosal and cutaneous leishmaniasis patients [6]. IL-17 and neutrophils have been associated with pathology in mice infected with *L. braziliensis* and in human leishmaniasis [7,8]. We have previously shown impairment of the ability of the regulatory cytokine IL-10 to downmodulate inflammatory cytokine production in cutaneous and mucosal leishmaniasis [9]. However, the role of other cytokines in modulating IFN-γ and TNF-α remains to be determined. TGF-β is known to decrease lymphocyte proliferation and cytokine production [10–12]. While IL-27 may induce lymphocyte activation during the initial steps of the immune response [13], it has an important regulatory function to prevent inflammation and subsequent tissue damage in the late phase of the immune response [14].

In the majority of diseases in which type 1 immune responses play a role in pathology, the key cytokines are TNF-α and IFN-γ [15]. In CL and ML, although antimony therapy failure occurs in up to 35% of cases [16] the use of pentoxifylline, a TNF-α inhibitor that modulates IFN-γ production, has resulted in an increased rate of cure. Moreover, pentoxifylline plus antimony therapy reduces the healing time of cutaneous and mucosal ulcers and cures CL and ML patients that are refractory to antimony treatment alone [17–19]. Therefore, appropriate modulation of the immune response play a key role in decreasing the pathology associated with the exaggerated Th1 immune response observed in human leishmaniasis.

The aim of the present study was to better understand the cytokine network during *L. braziliensis* infection and to identify molecules associated with potentiating of the inflammatory response or downregulating the inflammatory response. The identification of target molecules that may downregulate the inflammatory response may help to attenuate pathology in cutaneous and mucosal leishmaniasis.

## 2. Materials and methods

### 2.1. Study population

Forty CL patients and eighteen ML patients recruited at the health center of Corte de Pedra, an endemic area of *L. braziliensis* transmission that is located in the state of Bahia, Brazil, were enrolled in this study. Only those CL patients with a single typical ulcerative skin lesion of 1–3 months in duration without evidence of mucosal involvement and without a history of previous therapy were enrolled in this study. Mucosal leishmaniasis patients presented lesions in nasopharyngeal regions. The diagnosis of CL and ML was performed by detecting parasites from culture aspirates or histopathology or by the presence of a typical CL or ML lesion plus a positive delayed-type hypersensitivity (DTH) reaction to a *L. braziliensis* antigen [20]. All participants provided informed consent, and the study followed the guidelines of the Ethical Committee of the Federal University of Bahia.

### 2.2. Cell cultures

Peripheral blood mononuclear cells (PBMC) were separated from heparinized venous blood by Ficoll-Hypaque gradient centrifugation. The cells were cultured in RPMI 1640 (Life Technologies GibcoBRL, Grand Island, NY, USA) containing 10% human AB serum (Sigma, St. Louis, MO, USA), HEPES and antibiotics at a concentration of 3 × 10^6^ cells/mL These cells were plated in 24-well flat bottom microtiter plates (Falcon; Becton Dickinson, Lincoln Park, NJ, USA) and stimulated with media alone (unstimulated), 5 μg/mL of soluble *Leishmania* antigen (SLA) or with SLA and other specific stimuli. To determine whether IL-27 downregulates IFN-γ and TNF-α production as well as to determine its effect on IL-10 production, recombinant human IL-27 (rIL-27) (R&D Systems, Minneapolis, MN, USA) was added to PBMC cultures at 100 ng/mL To evaluate whether the neutralization of IFN-γ decreases TNF-α, anti-human IFN-γ (R&D Systems, Minneapolis, MN, USA) was added to PBMC cultures at a concentration of 100 μg/mL The results neutralizing TNF-α on IFN-γ production was evaluated by adding anti-human TNF-α (R&D Systems, Minneapolis, MN, USA) at 20 μg/mL to PBMC cultures. To determine if TGF-β altered TNF-α and IL-17 production recombinant human TGF-β (rTGF-b1, 10 ng/mL) (R&D Systems, Minneapolis, MN, USA) were added to PBMC cultures. The concentrations of cytokines or monoclonal antibodies added to the cultures were determined after a dose-response curve was generated in a limited number of patients and the optimal concentration was chosen for suppression experiments (data not shown).

### 2.3. Cytokine production

Cell cultures were incubated at 37 °C with 5% CO_2_ for 72 h or 96 h in the case of measuring IL-17 levels. IFN-γ, TNF-α, IL-10 and IL-17 levels were determined in supernatants using the sandwich ELISA technique (BD Bioscience Pharmingen, San Jose, CA, USA). The results are expressed in pg/mL.

### 2.4. Statistical analysis

Statistical analysis was performed with the Wilcoxon matched pairs test using GraphPad Instat Software (GraphPad, San Diego, CA, USA) and GraphPad Prism 5 Software (GraphPad, San Diego, CA, USA). The results are expressed as medians (interquartile range). Differences were considered to be statistically significant when *P* ⩽ 0.05.

## 3. Results

### 3.1. Clinical and immunological characteristics of cutaneous leishmaniasis patients (CL) and mucosal leishmaniasis patients (ML)

As demonstrated in Table 1, there were no statistical differences in age, gender, DTH, TNF-α, IL-17 and IL-10 levels between the two groups (all > 0.05). However, the mean of IFN-γ was found to be significantly higher in the mucosal leishmaniasis patients (*p* < 0.05).

### 3.2. IL-10 and TGF-β downregulate TNF-α and IL-17 production by cells from leishmaniasis patients

**Fig. 1:**
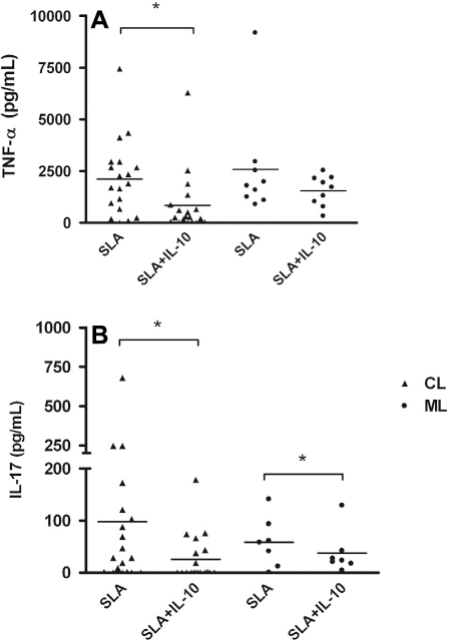
Modulator activity of IL-10 in the production of TNF (A) and IL-17 (B) by cells from patients with tegumentary leishmaniasis: PBMC from patients with CL and ML were stimulated with SLA (5 μg/ml) in the presence or not of IL-10 (10 ng/ml). The production of TNF (A) and IL-17 (B) was evaluated by ELISA. Statistical analysis was performed by Wilcoxon matched pairs test. (*p < 0.05).

We have already shown a decreased ability of IL-10 to down-regulate IFN-γ in patients with ML [9]. The ability of IL-10 and TGF-β to downregulate TNF-α and IL-17 production is shown in Fig. 1. IL-10 downregulated TNF-α production in CL patients but not in ML patients (Fig. 1a). In cells from CL patients, the median level of TNF-α in cells stimulated with SLA was 1893 pg/mL (0–7440 pg/mL), while stimulation with SLA and IL-10 resulted in TNF-α levels of 280 pg/mL (0–6280 pg/mL) *P* < 0.0005. In cells from ML patients, the concentration of TNF-α upon stimulation with SLA was 1790 pg/mL (892–9173 pg/mL) and stimulation with SLA and IL-10 did not decrease the level of TNF-α production, which was 1715 pg/mL (320–2526 pg/mL) (*P* > 0.05). IL-10 also had an inhibitory effect on IL-17 production in cells from both CL and ML patients (Fig. 1b). In cells from CL patients stimulated with SLA, IL-17 levels were 28 pg/mL (0–680 pg/mL) and decreased to 0 pg/ml (0–178 pg/mL) in cells stimulated with SLA plus IL-10 (*P* < 0.05). In cells from ML, IL-10 also decreased IL-17 production in culture stimulated with SLA from 58 pg/mL (0–141 pg/mL) in the absence of IL-10 to 23 pg/mL (4–129 pg/mL) after addition of IL-10 (*P* < 0.05).

**Fig. 2:**
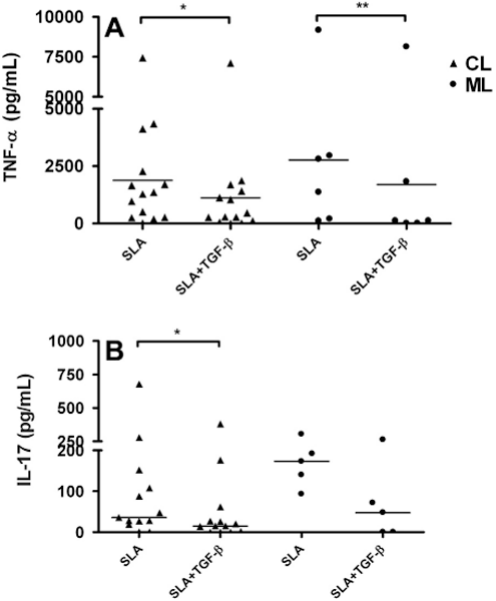
Modulator activity of TGF-β in the production of cytokines by cells from patients with tegumentary leishmaniasis: PBMC from patients with CL and ML were stimulated with SLA (5 μg/ml) in the presence or not of TGF-β (10 ng/ml). The production of TNF (A), IL-17 (B) and IFN-γ (C) was evaluated by ELISA. Statistical analysis was performed by Wilcoxon matched pairs test. (*p < 0.05).

The activity of TGF-β was assessed to examine its effect on the production of TNF-α and IL-17. TGF-β decreased TNF-α levels from 1320 pg/mL (15–7440 pg/mL) to 367 pg/mL (0–7100 pg/mL) in cells from CL patients (*P* < 0.0005) and from 2080 pg/mL (110–9173 pg/mL) to 117 pg/mL (0–8127 pg/mL) in cells from ML patients (*P* < 0.05) (Fig. 2a). The addition of TGF-β to the culture decreased IL-17 production from 36 pg/mL (1–680 pg/mL) to 15 pg/ mL (0–382 pg/ml) in cells from CL patients (*P* < 0.005) but had no effect in cells from ML patients, as IL-17 production was 173 pg/mL (93–305 pg/mL) following SLA treatment and 48 pg/mL (0– 263 pg/mL) following SLA plus TGF-β (Fig. 2b) (*P* > 0.05).

IL-27 was not able to suppress TNF-α production by cells from leishmaniasis patients: In addition to its role in stimulating a Th1-type immune response, IL-27 is known to attenuate inflammatory cytokine production [21,22]. In the present study, IL-27 did not suppress IFN-γ production in cells from CL patients, which was 2203 pg/mL following SLA stimulation (105–12,560 pg/mL) and 2670 pg/mL (0–9150 pg/mL) following SLA plus IL-27 stimulation (*P* < 0.05). IL-27 also failed to downregulate the secretion of this cytokine in cells from ML patients, as IFN-γ levels were 2370 pg/ mL (0–24,880 pg/mL) following SLA stimulation and 1785 pg/mL (65–13,106 pg/mL) following SLA plus IL-27 stimulation (*P* > 0.05). Moreover, the addition of IL-27 did not change TNF-a production from cultured cells (data not shown).

IL-27 has been shown to induce IL-10 production by human CD4^+^ T cells [23]. In the present study, the addition of IL-27 to PBMC from CL and ML patients did not enhance IL-10 production by cells from CL patients, as the observed IL-10 production was 84 pg/mL (2–925 pg/mL) following SLA stimulation and 98 pg/mL (10–864 pg/mL) following SLA and IL-27 stimulation. Similar results were observed using cells from ML patients, as IL-10 levels were 43 pg/mL (8–130 pg/mL) following SLA stimulation and 77 pg/mL (32–308 pg/mL) following SLA plus IL-27 stimulation (*P* > 0.05).

### 3.3. Neutralization of TNF-α inhibits IFN-γ production and does not induce IL-10 production

**Fig. 3:**
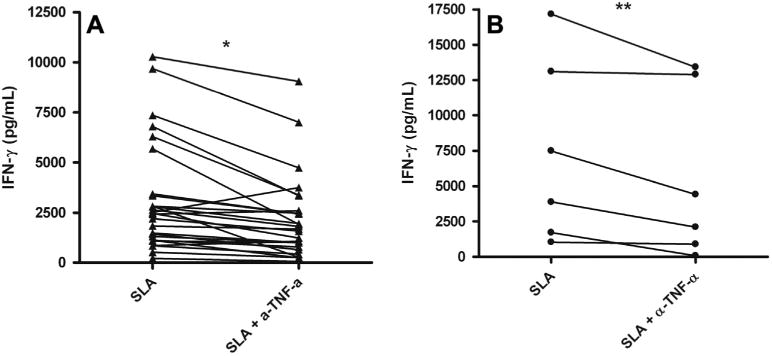
Neutralization of TNF-α affect IFN-γ production by cells from patients with tegumentary leishmaniasis: PBMC from patients with CL and ML were stimulated with SLA (5 μg/ml) in the presence or not of anti-TNF antibody (20 μg/ml). The production of IFN-γ was evaluated by ELISA. Statistical analysis was performed by Wilcoxon matched pairs test. (*p < 0.05; **p < 0.0005).

We have already shown that macrophages contribute to the production of TNF-α in human tegumentary leishmaniasis [9] and that this cytokine can activate of antigen presenting cells to thereby enhance the ability of CD4^+^ T cells to produce IFN-γ. To assess this cytokine production network, we stimulated cells from CL and ML patients with SLA in the presence or absence of an anti-TNF-α monoclonal antibody (mAb) and measured the production of IFN-γ (Fig. 3). Neutralization of TNF-α downregulated IFN-γ production from 2820 pg/ml (1120–10,280) following SLA treatment alone to 2440 pg/ml (250–9050) following mAb addition in cells from CL patients (Fig. 3a), *p* < 0.005. Also, TNF-α neutralization reduced IFN-γ production from 5687 pg/ml (1040–17,160) following SLA stimulation to 3255 pg/ml (80–13,420) following SLA plus mAb stimulation in cells from ML patients (Fig. 3b), *p* < 0.05. Neutralization of TNF-α did not modify IL-10 production (data not shown).

### 3.4. Neutralization of IFN-γ inhibits TNF-α production and induces IL-10 production

**Fig. 4:**
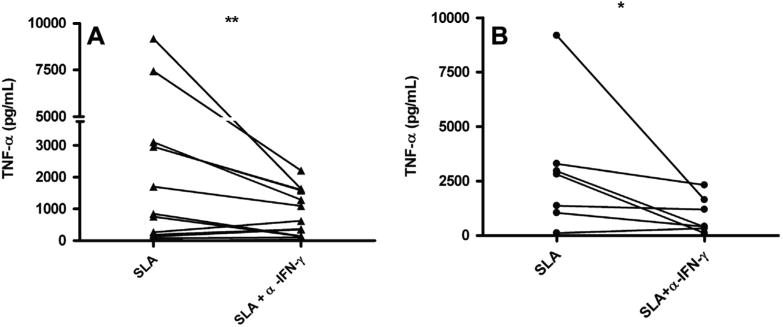
Neutralization of IFN-γ affect TNF-α production by cells from patients with tegumentary leishmaniasis: PBMC from patients with CL (A) and ML (B) were stimulated with SLA (5 μg/ml) in the presence or not of anti-INF-γ antibody (20 μg/ml). The production of TNF-α was evaluated by ELISA. Statistical analysis was performed by Wilcoxon matched pairs test. (*p < 0.05).

**Fig. 5:**
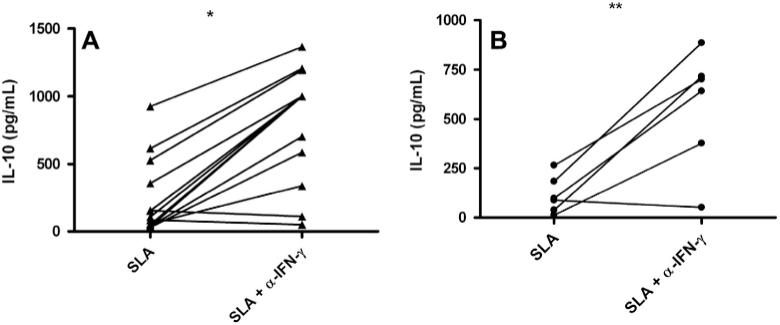
Neutralization of IFN-γ increase IL-10 production by cells from patients with tegumentary leishmaniasis: PBMC from patients with CL (A) and ML (B) were stimulated with SLA (5 μg/ml) in the presence or not of anti-INF-γ antibody (20 μg/ml). The production of IL-10 was evaluated by ELISA. Statistical analysis was performed by Wilcoxon matched pairs test. (*p < 0.005; **p < 0.05).

As the overproduction of IFN-γ could be involved in the high level of TNF-α production, we investigated the effect of neutralizing IFN-γ on TNF-α production by cells from patients with CL and ML. Neutralization of IFN-γ decreases TNF-α production in cells from CL and ML patients (Fig. 4). In cultures stimulated with SLA, the median level of TNF-α production was 802 pg/mL (0–9173 pg/mL), while the addition of anti-IFN-γ resulted in a TNF-α level of 494 pg/mL (0–2210 pg/mL) in cells from CL patients (Fig. 4a), *P* < 0.05. In cells from ML patients, the level of TNF-α production was 2800 pg/mL (110–9173 pg/mL) without anti-IFN-γ and 401 pg/mL (100–2309 pg/mL) after IFN-γ neutralization (Fig. 4b), *P* < 0.05. The neutralization of IFN-γ increased IL-10 production as well. In cells from CL patients, the median level of IL-10 production was 107 pg/mL (31–925 pg/mL) and after neutralization of IFN-γ, the level of IL-10 was 1001 pg/mL (51–1365 pg/mL). In cells from ML patients, the IL-10 level was 92.5 pg/mL (12–265 pg/mL) when cells were stimulated with SLA and 671 pg/mL (51–886 pg/ mL) after neutralization of IFN-γ, *P* < 0.005 and *P* < 0.05, respectively (Fig. 5a and b).

## 4. Discussion

In this study, we have demonstrated that the modulation of the inflammatory response in patients with tegumentary leishmaniasis caused by *L. braziliensis* is associated with increased production of IL-10. There are multiple studies that have shown that a strong Th1-type immune response including the production of the proinflammatory cytokines IFN-γ and TNF-α and decreases in production of IL-10 or the IL-10 receptor leads to an exaggerated inflammatory response that is responsible for lesion development in CL and ML patients [9,24,25]. In this study, it was shown that while IL-10 and TGF-β modulate the production of TNF-α and IL-17, IL-27 did not have an important role in modulating the production of inflammatory cytokines. Moreover, we showed that IFN-γ plays an important role in the maintenance of inflammation as neutralization of IFN-γ decreased TNF-α production and increased the production of IL-10.

IL-10 is known to have anti-inflammatory proprieties and to be necessary to control the strong inflammatory response that may follow a tissue immune response [26]. IL-10 play a key role in downregulating IFN-γ production in human visceral leishmaniasis [27–29], but studies have shown that this cytokine does not alter production of IFN-α in CL and ML patients [9]. In this study, we have shown that IL-10 can modulate the production of TNF-α by cells from CL patients, resulting in 85% suppression, but that this cytokine does not have the same effect on cells from ML patients. The regulatory activity of IL-10 depends on the expression of its receptor [30]. Previous studies have shown that ML patients produce more TNF-α and less IL-10 compared with CL patients [9]. One possible explanation for the decreased ability of IL-10 modulate the production of TNF-α in ML patients is the observation that cells from mucosal lesions express less IL-10 receptor when compared to cells from cutaneous lesions [24].

An important finding in this study is the observation that IL-10 decreases the production of IL-17 by cells from CL and ML patients. Although IL-17 is involved in the defense against several intracellular agents [31,32], this cytokine contributes to inflammation by inducing the recruitment of neutrophils and the production of various inflammatory mediators such as IL-1, IL-6, and TNF-α [33]. PBMC from patients with CL and ML produce more IL-17 than do cells from uninfected individuals, and this cytokine is also present in the lesions of these leishmaniasis patients [6,8], indicating that it may participate in the tissue destruction observed in this disease. The observation that IL-10 decreases the production of IL-17 by cells from CL and ML patients supports other studies that show that IL-10 can regulate IL-17 production in other inflammatory diseases such as colitis and chronic pulmonary *Mycobacterium avium* infection.

TGF-β is another cytokine that is able to regulate the inflammatory immune response by suppressing the differentiation of CD4^+^ effector cells, inducing the conversion of naïve T cells into Treg cells, inhibiting the production of IL-2 and IFN-γ and suppressing the activity of macrophages, dendritic cells and natural killer (NK) cells [34]. In a human cutaneous leishmaniasis system, the *in vitro* addition of TGF-β to cultures of PBMC from ML and CL patients did not inhibit IFN-γ production in response to leishmania antigen but did inhibit the production of IFN-γ by cells from healthy individuals stimulated with PPD by 47% [9]. In this study however, TGF-β abolished the TNF-α production by cells from patients with CL and ML. The inhibitory effect of TGF-β on Th1 cells is minimal in activated T cells, where there is decreased expression of TGF-β receptors, and this expression is IL-10 dependent [35]. The previous observation that the levels of IFN-γ are greater than the levels of TNF-α in these patients and that the production of IL-10 is low or absent in these patients [9] may explain why TGF-β has a greater effect on the production of TNF-α than on IFN-γ production in patients with CL. Although controversial, it has been documented that IL-6 and TGF-β induce the production of IL-17 by naïve T cells [33]. However, the results presented in this study suggest that TGF-β may be a modulator of IL-17 production in cells from patients with CL. As the cells from these patients have previously been activated and are being restimulated *in vitro* by leishmania antigen, TGF-β may have more of a modulatory role than a role as an inducer of IL-17 in this system.

Initial studies of the biology of IL-27 provide evidence for the role of this cytokine in initiating the Th1-type immune response [13,36]. However, subsequent studies have indicated that IL-27 has a wide inhibitory role on Th1 cells, preventing the immunopathology caused by this class of T cells [14,37]. Our results indicate that IL-27 does not affect the production of IFN-γ, TNF-α or IL-17 by the cells from patients with CL and ML. The anti-inflammatory effects of IL-27 are partially due to production of IL-10 [23,38]. The addition of IL-27 to PBMC cultures from patients with CL and ML did not interfere with the production of IL-10 by these cells, confirming that IL-27 did not have an effect on regulating the strong inflammatory response observed in human cutaneous leishmaniasis in this study.

It has been observed that macrophages from patients with CL and ML that are infected with *L. braziliensis* produce large amounts of TNF-α when compared with macrophages from individuals with subclinical infection by *L. braziliensis* [39]. As this initial production of TNF-α by macrophages during the innate immune response could contribute to the exaggerated production of IFN-γ from the T cells of these patients, neutralization of TNF-α could interfere with the secretion of IFN-γ. In this study, it was observed that the inhibition of TNF-α diminished the production of IFN-γ, but did not interfere with the production of IL-10 by PBMC from patients with ML and CL. These results together with the initial data that demonstrated that treatment with antimony associated with pentoxifylline, an inhibitor of TNF-α, cures patients refractory to antimony and accelerates the healing of these patients [18,19] suggest that TNF-α may contribute to the exaggerated production of IFN-γ and tissue destruction observed in this disease.

However, the inhibition of IFN-γ significantly decreased the production of TNF-α by PBMC from patients with CL and ML. Thus, the production of IFN-γ by Th1 cells could also stimulate the production of TNF-α by these cells. The decrease of TNF-α observed in this study was accompanied by an increase in the production of IL-10 by these patients. This finding is important as IL-10 has been shown have a protective role in some inflammatory diseases [38,40,41].

In conclusion, together, these results indicate that in tegumentary leishmaniasis caused by *L. braziliensis*, IFN-γ and TNF-α are the main cytokines involved in the tissue damage observed in this disease and that IL-10 may have a protective role. Further studies are needed to elucidate the rationale for use of IL-10 therapy in human tegumentary leishmaniasis.

## Figures and Tables

**Table 1:** Clinical and immunological characteristics of cutaneous leishmaniasis patients (CL) and mucosal leishmaniasis patients (ML).

	Cutaneous leishmaniasis (*n* = 40)	Mucosal leishmaniasis (*n* = 18)	*p* Value^a^
Age (years)	31.4 ± 13.7	38.3 ± 15.3	<0.05
Gender (% of male)	29 (72%)	9 (50%)	<0.05
DTH reaction to a *Leishmania* antigen (mm)	17 × 16	19 × 17	<0.05
IFN-γ (pg/ml)	3960 ± 729	5162 ± 1688	>0.05
TNF-α (pg/ml)	1695 ± 232	2285 ± 539	<0.05
IL-17 (pg/ml)	75 ± 23	98 ± 22	<0.05
IL-10 (pg/ml)	176 ± 36	78 ± 24	>0.05

Values are displayed as mean ± SEM; DTH: delayed-type hypersensitivity.

a Tests of significance between groups were based on Mann Whitney test.
